# The Hunterian Oration, Delivered in the Theatre of the Royal College of Surgeons in London, February 14, 1838

**Published:** 1838-07

**Authors:** 


PART SECOND.

### Bibliographical Notices.

---

Art. I.—*The Hunterian Oration, delivered in the Theatre of the Royal College of Surgeons in London, on the 14th of February*, 1838. By Benjamin Travers, f.r.s. &c.—*London*, 1838. 4to. pp. 42.

We are not surprised that Mr. Travers should have felt and expressed the difficulty of composing a fresh eulogium on the character and merits of John Hunter. Three-and-twenty times has this tribute to his memory been paid; and every succeeding writer must necessarily experience increased embarrassment in the selection of topics upon which to descant. As John Hunter, however, was neither anatomist, physiologist, surgeon, nor naturalist *alone*, but the most remarkable combination of all these which the world has yet seen, the occasion presents opportunity for considerable variety of panegyric; and it is natural that each writer should choose a subject most congenial to his own taste. Mr. Travers, therefore, directs our attention in this Oration to the peculiar claims of Hunter to eminence as a surgeon; and these he justly regards as not so much based on his improvement of the *art* of surgery as on the vast progress which he caused it to make as a branch of the *science* of pathology.

"The Hunterian era, the most memorable in the annals of our science, dates little earlier than the commencement of the present century. Shakspeare's *Marc Antony* says,

'The evil that men do lives after them;
The good is oft interred with their bones.'

If to Hunter any evil could be imputed, it was buried with him: his great achievements in the cause of our science, the proudest trophies of his fame, are posthumous. For, with the solitary but splendid exception of the operation for the cure of the popliteal aneurism, how imperfect a title would the most elaborate recital of his merits as a surgeon prove to the admiration and gratitude associated with his name.

"No; it is to his discoveries as an observer, sagacious, comprehensive, and profound, of the animal machine and its economy, in health and in disease,—his development of the phenomena which characterize inflammation in all textures, in its several aspects, stages, and processes, of the signs by which they are indicated, the laws by which they are governed, the consequences to which they lead, and the modes of treatment by which they are influenced and regulated, to subserve nature's and our purposes;—it is to these that we point with a national and honest pride, as to the column upon which are engraven in imperishable characters the surgical triumphs of John Hunter.

"He was not remarkable either for his skill as an operator, or his facility of communicating knowledge as a teacher. He was not a scholar; and neither the arrangement of his thoughts, nor the language in which they are clothed, is free from many and obvious exceptions. But the habitual character of his mind, which in the largest sense imparted itself to his works, was acutely observant, profoundly contemplative, capable of large and comprehensive views of natural phenomena, and endowed with a microscopic faculty of seizing and analyzing their constituent parts and bearings. Grant that his inductions did not always bear him out, that his combinations were sometimes inaccurately formed, and his announcement of general laws sometimes premature; yet where, in the calendar of time, shall we look for an equal in the compass, the variety, or the depth of his researches into the mysteries of animal life, or for consequences such as those that have resulted from his labours to universal pathology." (pp. 27-29.)

In order to demonstrate more clearly the actual improvements effected by Hunter, Mr. Travers has entered into an historical review of the progress of surgery from the time of Hippocrates, Galen, and Celsus, to the last century; and, were we inclined to be minutely critical, we might perhaps suggest that, with less of minute detail, the general ideas might have been more prominently brought forwards. We shall not, however, attempt to disparage the intrinsic excellence of the review; but shall conclude the present notice by directing the attention of our readers to another of those remarkable *anticipations* which the works of John Hunter are found to present, in proportion as the advance of science develops the ideas which appear to have almost intuitively suggested themselves to his extraordinary mind.

"Even of that sublime science of these our days, which connects the history of our planet with that of its extinct races, which has based their classification on the anatomical correspondence of uniformity of design, manifest in the organic remains of periods remotely antecedent to the creation of man and the existing types of mortality, it is indisputable that Hunter had a prophetic vision. For proof of this, I may refer, not only to his rich fossil collection, including at his decease about 1050 specimens, but to his interesting posthumous paper in the Philosophical Transactions for 1794, on the Fossil Bones found in the Caverns of the Principality of Bayreuth. In this paper he compares these specimens with their recent analogies, and shows that they differ both from them and among themselves.

"He alludes to the different climates and localities of the globe to which animals are more or less confined, or their geographical distribution, which, considered in relation to fossil remains, elucidates, by implication, the changes of temperature to which different parts of the earth have been subject at different periods.

"With more distinctness and detail, he points out the evidence which fossils afford of the alteration of the condition of the earth's surface, as dry land or submerged; and, by frequent allusion to the many thousand years which must have elapsed while the earth was the theatre of these changes, he seems to have fully appreciated the necessity of an ample allowance of past time to account philosophically for the changes in question. Mr. Clift, who transcribed the manuscript of this paper, informs me that it was originally dictated, not 'many thousand years,' but 'many thousand centuries;' and he preserves the copy of the letter from the friend of Hunter,* who advised the change of expression, in conformity with the popular notion of the world's age. Geologists now know that the latter expression is nearer the truth. I will only further remark, that, of three hypotheses which he suggests for the occurrence of the countless bony remains in the Gailenreuth caverns, he inclines to that which has been adopted by the distinguished Professor Buckland for the analogous collection in the caves of Kirkdale,—viz. that the caves were the habitual retreat of the extinct species while living; and that there they brought the carcases of their prey, and fed; and that there they came, and died.

"This interesting paper, if candidly perused, must, I think, be considered as the dawning of that glorious daylight with which fossil anatomy, the handmaid of geology, has since overspread the summits and penetrated the depths, and thus illumined the history of 'the earth, and of the waters under the earth.'" (pp. 33-35.)

* The late Major Rennell.

We cannot but regard this anecdote of Hunter as deserving to be placed on record by the side of Newton's anticipation of the combustibility of the diamond, from its highly refractive power.

